# The Electro-Therapeutist

**Published:** 1897-09

**Authors:** 


### The Electro-Therapeutist.

Every physician who uses electricity should send for a copy of *The Electro-Therapeutist*, a monthly journal devoted to electro-therapeutics for the general practitioner. Write the editor, Wm. F. Howe, M. D., Indianapolis, Ind., mentioning this journal, and he will send you sample copies gratis.

